# Assessing Fifty Years of General Health Surveillance of Roe Deer in Switzerland: A Retrospective Analysis of Necropsy Reports

**DOI:** 10.1371/journal.pone.0170338

**Published:** 2017-01-19

**Authors:** Mirjam Pewsner, Francesco Carlo Origgi, Joachim Frey, Marie-Pierre Ryser-Degiorgis

**Affiliations:** 1 Centre for Fish and Wildlife Health, Vetsuisse Faculty, University of Bern, Bern, Switzerland; 2 Institute of Veterinary Bacteriology, Vetsuisse Faculty, University of Bern, Bern, Switzerland; US Geological Survey, UNITED STATES

## Abstract

General wildlife health surveillance is a valuable source of information on the causes of mortality, disease susceptibility and pathology of the investigated hosts and it is considered to be an essential component of early warning systems. However, the representativeness of data from such surveillance programs is known to be limited by numerous biases. The roe deer (C*apreolus capreolus capreolus*) is the most abundant ungulate and a major game species all over Europe. Yet, internationally available literature on roe deer pathology is scarce. The aims of this study were (1) to provide an overview of the causes of mortality or morbidity observed in roe deer in Switzerland and to assess potential changes in the disease pattern over time; and (2) to evaluate the value and limitations of a long term dataset originating from general wildlife health surveillance. We compiled 1571 necropsy reports of free ranging roe deer examined at the Centre for Fish and Wildlife Health in Switzerland from 1958 to 2014. Descriptive data analysis was performed considering animal metadata, submitter, pathologist in charge, laboratory methods, morphological diagnoses and etiologies. Recurrent causes of mortality and disease pictures included pneumonia, diarrhea, meningoencephalitis, actinomycosis, blunt trauma, predation, neoplasms and anomalies. By contrast, other diagnoses such as fatal parasitic gastritis, suspected alimentary intoxication and reproductive disorders appeared only in earlier time periods. Diseases potentially relevant for other animals or humans such as caseous lymphadenitis (or pseudotuberculosis), salmonellosis, paratuberculosis and listeriosis were sporadically observed. The disease pattern in roe deer from Switzerland was largely in accordance with previous reports. The observed fluctuations were consistent with methodical and/or personnel changes and varying disease awareness. Nevertheless, despite such limitations, the compiled data provide a valuable baseline. To facilitate comparison among studies, we recommend systematically archiving all case documents and fixed tissues and to perform data analyses more regularly and in a harmonized way.

## Introduction

The need for wildlife health surveillance and its benefits to animal and human health and nature conservation are now widely recognized. In Europe, wildlife health surveillance programs have either already existed for a long time or else have recently been established in numerous countries. Nevertheless, only few of them have in place a general surveillance program, which is comprehensive with respect to species of animals examined and types of diseases assessed [1]. By definition health surveillance includes not only data collection on disease and pathogen occurrence but also analysis of the collected data for decision-making and policy formulation [2]. However, compilations of wildlife health data acquired through general surveillance in Europe, if at all existing, have been mainly written in the respective local language and have to date remained scarce in the internationally available literature. To obtain an overview of the health status of wildlife at a continental level, to assess differences between regions or countries, and understand the factors causing these differences, it is important to make existing data widely available.

The European roe deer (*Capreolus capreolus capreolus*) is the most common and abundant wild ungulate in Europe. It is considered originally a forest dwelling species but due to its digestive plasticity and ability to adapt to new habitats, it has successfully colonized the modern agriculture landscape and coped with the agricultural intensification and forest fragmentation [3,4]. A re-increase of forests and wild ruminant densities together with changes in wildlife and livestock management over the past century has resulted in increased interactions between wildlife, humans and domestic animals, leading to an increased risk of pathogen transmission among them [5]. The population of roe deer in Europe is nowadays estimated at 10 million individuals, distributed widely across various habitats ranging from Mediterranean to boreal areas [6]. Accordingly, the roe deer is one of the most important game species in Europe [7] and also an important prey species for wild carnivores [6]. Interactions additionally occur with domestic animals, mainly livestock on grazing pastures [8] and hunting dogs. The surveillance of the health status of roe deer is therefore not only relevant for the conservation of the species itself, but also for the health of other wildlife, domestic animals and humans. Furthermore, due to the roe deer’s wide distribution in diverse habitats, its health status may serve as an indicator for environmental health. Surprisingly, there exist only a few scientific articles in English language which compile roe deer health data originating from general surveillance (Sweden: Aguirre [9]; and France: Lamarque [10]). Other publications have indeed been written in the respective local language and were mostly published in earlier years (e.g. [11–14]). More recent data found in the international literature are restricted to targeted investigations, which either considered a wide range of pathogens [15–20] or focused on specific infections such as bovine virus diarrhea [8,21], bluetongue virus [22,23], *Babesia* sp.[24], *Ehrlichia* sp. [25], paratuberculosis [26–29], bovine tuberculosis [30] and Schmallenberg virus [31–33].

General wildlife health surveillance in Switzerland dates back to the 1950ies. Several institutions have been involved in this task but a unit (now called Wildlife Group within the Centre for Fish and Wildlife Health, FIWI) entirely dedicated to wildlife health has been part of the Veterinary Faculty of the University of Bern since 1956 and been mandated to carry out a comprehensive general health surveillance program for the whole country [34]. The roe deer nearly vanished from Switzerland at the end of the 19^th^ century, as a consequence of habitat destruction and overhunting. This situation concerned all other large wild mammals and caused the total extinction of most of them including the large predators (Eurasian lynx *Lynx lynx*, grey wolf *Canis lupus* and brown bear *Ursus arctos*). With the implementation of federal laws that protected forests and regulated hunting and the initiation of reintroduction programs, forests and population of wild mammals slowly recovered [35]. The roe deer population increased again through immigration from neighboring countries and the release of few animals of unknown origin at the end to the nineteenth century [36]. The Eurasian lynx, the main natural predator of roe deer, was reintroduced in several Swiss regions in the 1970’s [37] and since 1995 the grey wolf has also made a progressive come-back [35]. In parallel, urbanization including road networks has increased in lowland areas at the cost of agricultural land, while forests have regained ground in mountainous areas [36]. Nowadays the roe deer occupies almost any suitable habitat from the Swiss lowlands up to 2000m above sea level. About 40,000 animals are hunted and consumed annually and 8,000 die in traffic accidents every year [38]. At the national level the population has remained stable since 1980 [36] although locally decreasing densities have been of concern [39].

The present study had two main goals: (1) to provide an overview of the data obtained on diseases and other causes of death in roe deer in the framework of the general surveillance program at the University of Bern over half a century; (2) to explore and discuss the value and limitations of data derived from carcasses selected by field partners and submitted over such a long time period. The roe deer was a particularly good candidate to address this issue because: i) it is the only species which has been regularly submitted to the Wildlife Group in relatively high numbers since the earliest years; ii) it has been impacted by relevant environmental changes over the study period; iii) this species is widespread in the whole country as well as in the rest of Europe. We hypothesized that these environmental changes together with those concerning the field and laboratory personnel and the changes in the laboratory procedures might have contributed to a modification of the observed disease pattern over time.

## Material and Methods

### Ethics statement

This study did not involve purposeful killing of animals. All samples originated from dead wildlife (found dead in the field, legally shot because of severe debilitation or legally hunted during the official hunting season). According to the legislation of Switzerland (922.0 hunting law and 455 animal protection law, including legislation on animal experimentation; www.admin.ch) and the Principality of Liechtenstein (www.gesetze.li), no ethical approval or permit for animal experimentation was required.

### Study area

Switzerland is a small country of 41,285 km^2^ located in the middle of central Europe. It is divided into 26 political units of various sizes called cantons and includes three main bioregions: the Jura Mountains, characterized by forests and pastures; the highly urbanized Swiss Midlands; and the Alps, of which a large part is located above the timber line [40,41]. The Principality of Liechtenstein (160 km^2^) is adjacent to the eastern Swiss border and affiliated with the Swiss veterinary services (status similar to that of a canton). There are three different hunting regimes: the licensing hunting system (16 cantons or 70% of the Swiss national territory and the Principality of Liechtenstein), the preserve system (9 cantons) and prohibited hunting (one canton: Geneva). Officially appointed game wardens are present in all cantons with the licensing system and in Geneva [36]. In cantons with the preserve system, their tasks are partly performed by hunters (voluntary gamekeepers), except for one canton (St. Gallen), where professional game wardens are also present.

### Study material

Archived necropsy reports concerning 1674 free ranging roe deer examined by the Wildlife Group between 1958 and 2014 were available, and 1571 of them were considered for this study. Data were available for all years except for 1968 and 1980–82. The following cases were excluded because they had been submitted for other reasons than general health surveillance of free ranging wildlife: mammary glands sent for assessing the lactations status (n = 9); samples submitted for targeted investigations on selected pathogens relevant to human or animal health (rabies virus, foot and mouth disease virus or *Brucella* sp.; n = 10, all negative); carcasses and organs investigated in the framework of an interrupted animal experiment under field conditions (n = 24); hand raised fawns that died later than 3 days after arrival in captivity (n = 24); single organs without relevant pathological changes (n = 36).

### Laboratory methods

Full necropsies of all carcasses and gross examination of other submitted material were performed over the entire study period but the level of accuracy of the descriptions and diagnoses in the necropsy reports as well as the readability and completeness of the archived documents strongly varied over time. Five main pathologists were in charge of the necropsy duty (as primary investigators or supervisors) during the study period, which we divided accordingly into five time periods: (1) 1958–1985; (2) 1986–1994; (3) 1995–2000; (4) 2001–2009; (5) 2010–2014. During the periods 4 and 5, the main pathologists in charge were board-certified by the American College of Veterinary Pathology. At least from 1996 onwards, diagnostic of predation was performed according to known patterns of predator attacks and caused wounds as described by Molinari et al. [42]. In case of suspicion of predation by wolves, swabs were taken from bite wounds and consumed tissues to collect saliva samples for genetic analysis at the Institut d’Ecologie, Laboratoire de Biologie de la Conservation, Lausanne, Switzerland [43].

Tissues collected for histology were fixed in 10% buffered formalin, processed, embedded in paraffin, sectioned and stained with hematoxylin-eosin and other special stains as required according to standard protocols. Since 2001, standards for slide preparation have followed the accredited protocols of the Institute of Pathology of the University of Bern.

Parasitology, bacteriology and mycology methods applied in former times are largely unclear because they were not indicated in the reports or documented elsewhere. Bacteriological and mycological examinations were performed at the Institute of Veterinary Bacteriology of the University of Bern from 1956 to 1985, and have been performed again at this institute since 1997. Between 1985 and 1997, bacterial cultures were performed in-house by the Wildlife Group. As far as we know, bacterial identification has been carried out using standard biochemical strips (API 20 E/NE) and mycological identification using Sabouraud-Dextrose-Agar (SAB). Polymerase chain reaction (PCR) for detection of *Mycobacterium avium* subsp. *paratuberculosis* was used on four cases sampled in 2011 and 2012 and performed at the national reference laboratory (Institute of Veterinary Bacteriology of the University of Zurich).

Parasitological examinations have been performed at the Institute of Parasitology (IPA) of the University of Bern since 1992. Before 1992 they had been carried out by the parasitology laboratory of the Institute of Animal Pathology and consisted of the examination of intestinal washouts obtained during necropsy. Since 1992, conventional coprological analyses and parasitological identifications have been carried out according to the basic methodology described by Deplazes et al. [44], including flotation, sedimentation and Baerman technique as standard procedures. In-house investigations by the Wildlife Group were partly additionally performed in older times, including parasitological necropsies and scrapings of the gastric and intestinal mucosa. Overall the accuracy of parasite identification has strongly varied, reaching from the species level up to overarching taxa such as “gastrointestinal nematodes”. Quantitative information, if given, was either semi-quantitative or consisted of parasite counts.

Virological investigations included mainly rabies testing (n = 111) using the fluorescent antibody test (FAT), but also sporadic examinations for bovine viral diarrhea (BVD Antigen-ELISA, n = 2) or bovine leukemia virus (ELISA, n = 1) performed at the Institute for Veterinary Virology of the University of Bern. Bornavirus investigation (n = 1) was carried out by immunohistochemistry (nucleoprotein p40, phosphoprotein p24) at the Institute for Veterinary Pathology of the University of Zurich. In-house investigations for herpesviruses were performed in one case with lesions suggestive of malignant catarrhal fever and in one case with ocular lesions, using a consensus panherpes PCR [45]. The amplicons obtained by PCR were then sent for automatic sequencing to a private biotech company (Microsynth, Belgach, Switzerland). The obtained sequences were compared with the NCBI herpesvirus sequence database by BLAST (http://blast.ncbi.nlm.nih.gov/Blast.cgi).

Toxicology was performed on one case only. It was carried out at the experimental station of Liebefeld (Bern, Switzerland) for the detection of ammonium nitrate (negative result).

### Data management

Animal data, submitter information, case history, pathologist in charge and results of all analyses performed were entered into an Excel-database (Microsoft Excel 2010, Microsoft Corporation, Redmond, Washington, USA). Since 1976 local place up to coordinates have been documented, but in earlier years details concerning the geographic data did not go beyond the level of the cantons. Therefore, only the canton of origin could be used for analysis. Maps were designed with the free QGIS- Software (QGIS Development Team, 2012. Versions 1.8.0, 2.0.1 and 2.2.0; QGIS Geographic Information System, Open Source Geospatial Foundation Project, http://qgis.osgeo.org/). Due to the heterogeneity and lack of representativeness of the material, statistical analyses were not performed.

According to the age indicated on the report, the animals were classified into four categories as described by Aguirre et al.[9]: Fawns (<1-yr old), yearlings (1–2 yr-old), adults (>2–7 yrs old) and old adults (>7-yr-old). Based on the information on the reports, diagnoses were classified based on the etiology as described by Sieber et al.[46]: The “main diagnosis” was defined as the main cause of disease (in case of euthanasia or culling) or cause of death, and classified as infectious, noninfectious or undetermined. Undetermined diagnoses included cases categorized as “not-diagnostic” if the submitted material was not appropriate for analysis (wrongly selected organs or advanced autolysis) and as “unclear” if no diagnosis could be achieved despite appropriate material. If appropriate, factors believed to have played a predisposing role for the development of the main diagnosis were additionally indicated. Secondary findings were defined as lesions independent of the main diagnosis.

Reported bacteria, parasites and fungi were recorded according to the current nomenclature [47,48].

## Results

### Submitted material and performed laboratory analyses

Of the 1571 cases considered for this study, nearly 80% consisted of whole carcasses (n = 1227; versus 344 cases consisting of selected organs). Almost 50% were animals found dead (n = 767), followed by those culled due to disease signs (35%, n = 546) or hunted (3%, n = 41); in 218 cases the manner of death was not provided. Females and males were similarly represented (757 and 708, respectively) but 106 roe deer were of unknown sex. Regarding age, 60% were adults (n = 875), of which 14% (n = 123) were indicated as old individuals. Fawns represented nearly a third of the cases (n = 431) while yearlings were only 10% (n = 157) and the age was not indicated for 104 cases.

**Fig 1:**
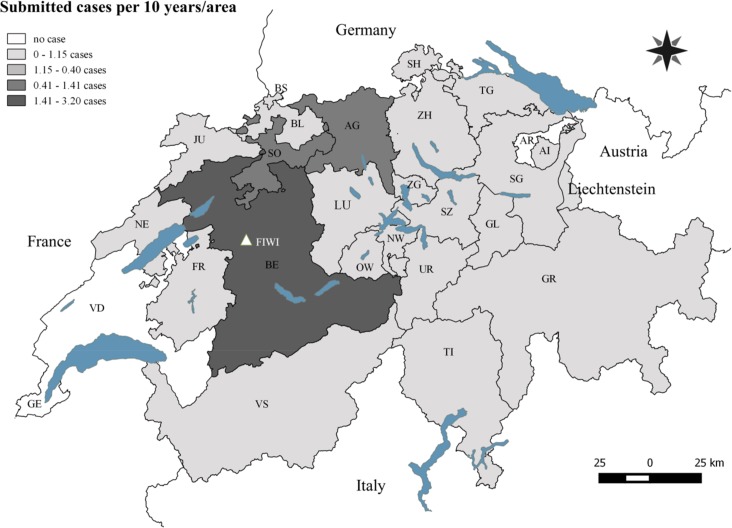
Map of Switzerland depicting the cantonal origin of the submitted roe deer. Shades of grey illustrate the number of roe deer per 10 years and 100km^2^ of cantonal surface. Abbreviations: AG = Aargau, AI = Appenzell Innerrhoden, AR = Appenzell Ausserrhoden, BE = Bern, BL = Basel-Landschaft, BS = Basel-Stadt, FR = Fribourg, GE = Geneva, GL = Glarus, GR = Graubünden, JU = Jura, LU = Luzern, NE = Neuchâtel, NW = Nidwalden, OW = Obwalden, SG = St. Gallen, SH = Schaffhausen, SO = Solothurn, SZ = Schwyz, TG = Thurgau, TI = Ticino, UR = Uri, VD, Vaud, VS = Valais, ZG = Zug, ZH = Zurich. FIWI = Centre for Fish and Wildlife Health (white triangle).

**Fig 2:**
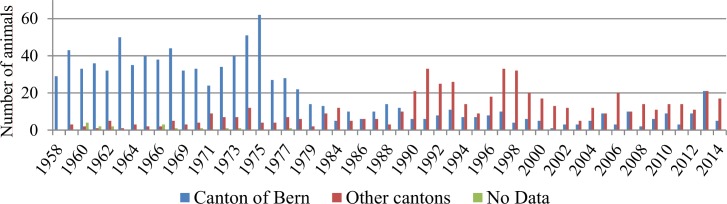
Number of submitted roe deer per canton and year. Slightly more than half of the cases were submitted by official game wardens (n = 893) followed by voluntary gamekeepers (n = 259). Considerably fewer cases were delivered by the police (n = 68), hunters (n = 57), private veterinarians or veterinary authorities (n = 48), and cantonal hunting authorities (n = 37). Overall, cantons with official game wardens submitted more animals (78%; n = 1227) than cantons without game wardens (20%; n = 313).

The animals were submitted from 23 out of 26 Swiss cantons and from the Principality of Liechtenstein. The large majority of the submissions originated from the canton of Bern (62%). Other cantons were far less represented, starting with Aargau (7%), followed by Solothurn (4%), St.Gallen (4%), Luzern (4%), Graubünden (4%) and Freiburg (3%; Fig 1). The other cantons and the Principality of Liechtenstein submitted less than 35 roe deer cases over the whole study period and the geographic origin was unknown in 3.7% of the cases. The number of submitted cases per 10 years and cantonal surface are shown in Fig 1. The predominance of submissions from Bern was present throughout the entire study period but more pronounced in the early years 1958–1985, when 90% of the submitted cases came from this region (Fig 2).

**Fig 3:**
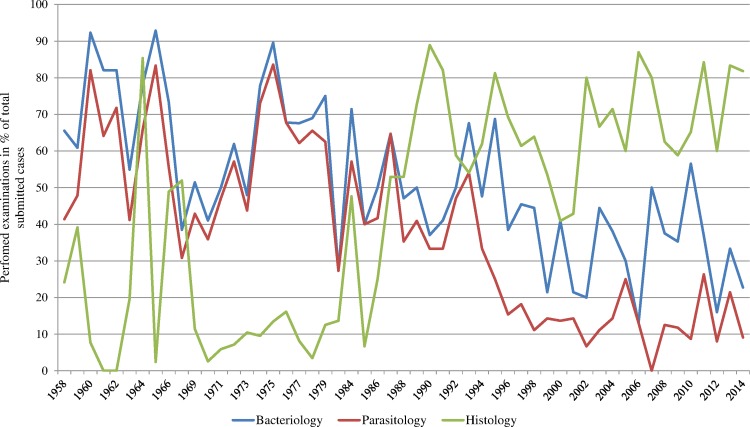
Performed parasitic, bacteriological and histological investigations over time.

Ancillary examinations that complemented gross pathology included parasitology (64% of the submitted cases; n = 1004), bacteriology (57%; n = 900), histology (42%; n = 662) and, less commonly, virology (8%; n = 122). Their frequency was marked by major fluctuations, but overall bacteriology and parasitology analyses were more often carried out in the early years while histology became progressively more common in recent years (with the exception of one early peak of histological examinations in the 70ies; Fig 3).

### Reported causes of mortality and morbidity

An overview of the etiologies of the main diagnoses is given in Table 1. They were more frequently infectious (46%) than noninfectious (39%). No diagnosis could be achieved in 15% of the cases. Reported infections were mainly attributed to bacteria (60% of infections) followed by parasites (27%), fungi (5%) and viruses (1%).

**Table 1:** Overview of the causes of mortality and morbidity diagnosed in free-ranging roe deer submitted to the Centre for Fish and Wildlife Health, Switzerland, 1958–2014.

	Adult			Juvenile	Fawns	No data		
	M	F	Total^1^				TOTAL	%
***Infectious***	217	192	361	82	182	44	720	46
**Bacterial**	144	105	219	39	119	25	433	28
Pneumonia	44	36	80	15	60	14	169	11
Meningoencephalitis	45	5	50	4	5	1	60	4
Actinomycosis or actinomycosis-like lesions	7	14	19	1	1	0	23	1
Miscellaneous	48	50	70	19	53	10	181	12
**Mycotic**	17	6	24	7	5	2	38	2
Pneumonia	9	5	15	2	2	1	20	1
Actinomycosis-like lesions	7	1	8	2	2	1	13	1
Miscellaneous	1		1	3	1	0	5	>1
**Parasitic**	40	63	103	32	45	14	194	12
Gastrointestinal	17	27	44	9	19	3	75	5
Pulmonary	5	6	11	6	9	5	31	2
Multisystemic endoparasitism	14	22	36	15	14	4	69	4
Miscellaneous	4	8	12	2	3	2	19	1
**Viral**	0	5	5	0	0	2	7	<1
**Undetermined infectious etiology**	16	13	30	4	13	1	48	3
***Non-infectious***	139	184	323	57	196	31	607	39
**Traumas**	88	101	189	37	127	18	371	24
Traffic accident	45	40	85	13	34	5	137	9
Agriculture machines	1	1	2	1	14	0	16	1
Intra-species fights	5	2	7	0	1	1	9	1
Predation	16	42	58	12	63	9	142	9
Gun wounds	15	10	25	8	9	2	44	3
Miscellaneous	6	6	12	3	6	1	22	1
**Orphans**	0	0	0	0	25	0	25	2
**Myopathies**	3	6	9	1	3		13	1
**Suspected physical exhaustion**^**2**^	17	11	28	6	8	1	43	3
**Neoplasms**	4	21	25	2	1	5	33	2
**Winter starvation**	1	8	9		9		18	1
**Alimentary disorders**	13	10	23	9	14	4	50	3
**Malformations**	5	4	9	2	5	2	17	1
**Degenerative diseases**	5	17	22	0	0	0	22	1
**Miscellaneous**	3	6	9	1	2	1	10	1
***Undetermined***	31	53	85	7	43	6	140	9
***Not diagnostic***	20	31	55	11	14	23	103	7
Total	407	460	875	157	435	104	1571	100

Numbers refer to the main diagnoses, i.e. the disease processes considered as cause of death of animals found dead and main cause of disease in shot animals, according to the age class (Adult, > 2-yrs-old; Fawn, < 1-yr-old). M, male; F, female.

^1^Including animals of unknown sex.

^2^35 suspected dog chases, 6 drowning, 2 others.

#### Bacterial and fungal infections

Bacterial infections were often inferred on the basis of the pathological changes but only 155 out of 433 (36%) were confirmed by microbiology, either because microbiological investigations were not performed or because the culture results were negative or inconclusive. Bacterial pneumonia was recurrently considered as cause of death throughout the study period, and when the diagnosis was supported by a conclusive bacteriological examination, the most commonly isolated bacteria were pyogenic organisms such as S*treptococcus* sp., *Staphylococcus* sp., *Trueperella* sp. in pure cultures or in mixed infections, *Pasteurella* sp. *(P*. *multocida*, *P*. *septica)* or *Manheimia* sp. (*M*. *haemolytica*, *M*. *granulomatis*, *M*. *varginea*). Lung parasites were considered to be a predisposing factor in 21% of the bacterial pneumonia cases.

**Fig 4:**
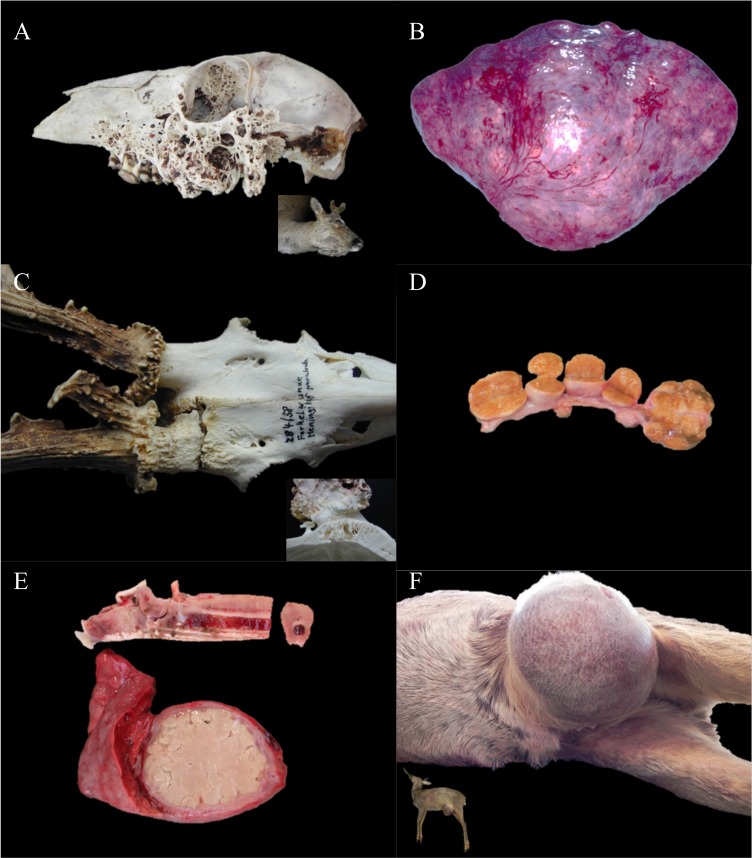
Selected gross pathology findings in free-ranging roe deer from Switzerland. (A) Skull, actinomycosis-like lesions of bacterial origin. A severe proliferative and destructive process is affecting the lateral aspect of the skull. Inset: The right mandibular region is severely expanded by a proliferative inflammatory process similar to that shown in the main figure (both cases were from 2013). (B) Spleen, lymphoma. There is diffuse and severe splenomegaly with multiple nodules expanding within the parenchyma and elevating the capsule. Additional similar lesions were also observed in liver, bone marrow and lymph nodes (1994). (C) Skull, osteomyelitis secondary to traumatic injury. A transverse fracture is associated with a granular appearance of the bone consistent with remodeling secondary to osteo-myelitis. Obvious bone loss and rarefaction is seen in cross section (inset) (1958). (D) Mesenteric lymph nodes, paratuberculosis. The mesenteric lymph nodes are severely enlarged (lymph-adenomegaly), up to 2–4 x 1.5–1 x 2–2.5 cm (2011). (E) Lung, tarsal and metatarsal bones, pyogranulo-matous fungal pneumonia and hypertrophic osteopathy. A large pyogranuloma is expanding, compressing and replacing the lung parenchyma. The cortical bone of the frontal aspect of the metatarsus is diffusely and markedly thickened by a bony proliferation (2010). (F) Inguinal hernia. A large mass covered by normal skin is bulging in the region of the left inguinal space (diameter 46cm). The mass contained small intestine loops and part of the caecum (2011).

Infected wounds, especially in the head region, were regularly reported throughout the study period. Suppurative meningoencephalitis (n = 60) was observed in both males and females. However, it was overrepresented in adult males (75%, n = 46) examined between April and October, with a peak in August (39% of the affected adult males). Frontal head injuries (suspected intra-species fights) and subsequent chronic skull damages were indicated as preliminary cause (n = 41) (Fig 4C). By contrast, in affected females the inflammatory process was mostly located at the brain base or in the region of the ethmoid and the data did not suggest any seasonal pattern. In two roe deer (one female and one male) with a purulent meningoencephalitis determined to originate from the base of the brain, tissue damage caused by botfly larvae was interpreted as the likely port of entry for pathogens. When bacteriology was performed, *T*. *pyogenes* was the bacterium most commonly isolated in males and *Pasteurella* sp. in females.

Actinomycosis and actinomycosis-like lesions consistent with proliferative mandibular or upper jaw osteomyelitis and cellulitis associated with the presence of “sulfure granules” were also repeatedly observed (n = 21) (Fig 4A). The most commonly isolated organism from these lesions was *Trueperella pyogenes* alone or in mixed infection (n = 5), whereas *Actinomyces* sp. was isolated in only two cases. Predisposing causes identified for these infections were gingival microtraumas including teeth damages in two old animals and botfly larvae in the nasal cavity of two other roe deer.

**Fig 5:**
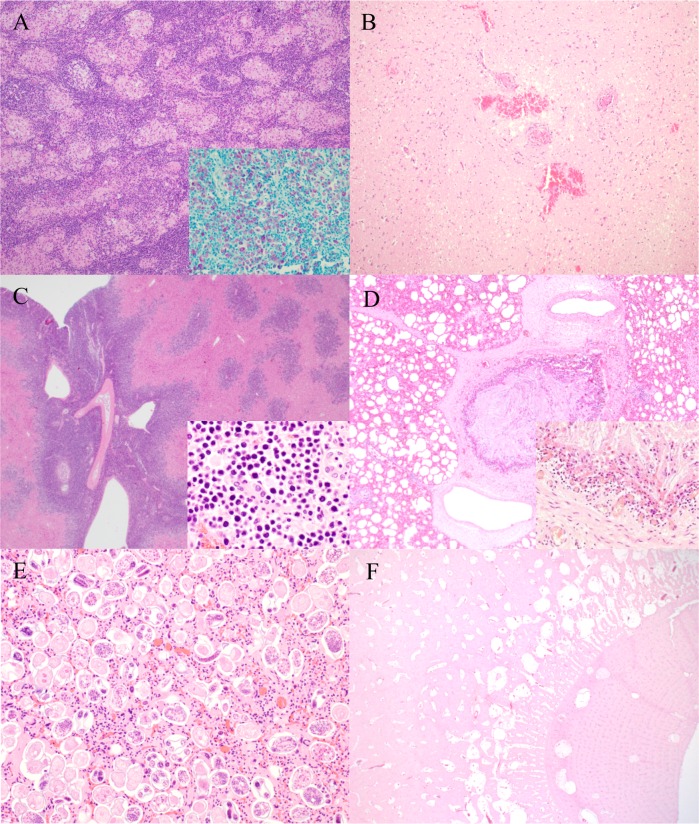
Selected microscopic pathology findings in free-ranging roe deer from Switzerland. (A) Mesenteric lymph node, paratuberculosis. There are large clusters of macrophages scattered in the lymph node parenchyma. Hematoxylin and eosin. Cells contain large numbers of intracytoplamatic acid fast bacteria (Inset). Ziehl Neelsen. (B) Brain, malignant catarrhal fever. Multifocal hemorrhages often associated with variably extensive clear spaces (edema) and hypereosinophilic neurons with glassy appearence (necrotic neurons) are present in the neuropil. There is multifocal fibrinoid vascular necrosis with weak mononuclear cuffing. Prominent syncytial cells are seen in the affected vessels (Inset). Hematoxylin and eosin. (C) Liver, lymphoma. Multifocal to coalescent sheets of neoplastic cells are infiltrating the liver parenchyma. The neoplastic lymphoid cells have large basophilic nuclei with small amounts of cytoplasm and show occasional mitoses (inset). Hematoxylin and eosin. (D) Lung, acute capture myopathy. The lumen of a bronchiole is completely obscured by large amounts of mucous. The wall of the bronchus is diffusely infiltrated by often degranulating globular leukocytes, associated with hyperemic vessels surrounded by clear spaces (edema) (Inset). Hematoxylin and eosin. (E) Lung, severe lungworm infestation. The alveolar spaces are diffusely replaced by embryonated nematode eggs. Eggs contain either morulas with a variable number of cells or variably developed larvae. (F) Metatarsal bone, hypertrophic osteopathy. A thick layer of proliferating bone is expanding radially from the cortical bone. Hematoxylin and eosin.

Moreover, sporadic cases of bacterial diseases associated with well-known clinical entities were observed. Generalized dermatophilosis (*Dermatophilus congolensis*) characterized by dark, thick, smeary crusts and partial hair loss was diagnosed in three fawns found in different regions and in different years. Yersiniosis caused by *Yersinia pseudotuberculosis* was reported five times over the study period. All cases presented a pneumonia, associated with multiple necrotic foci and abscesses in the lung and associated tracheobronchial (n = 1) or mesenteric lymph nodes (n = 1). Salmonellosis was diagnosed in two fawns (n = 1 *Salmonella* Typhimurium; n = 1 *Salmonella* sp.) with pneumonia as the main lesion. Two fawns with diarrhea were found to be infected with *Erysipelothrix rhusiopathiae*. Caseous lymphadenitis (*Corynebacterium pseudotuberculosis*) characterized by typical miliary tan nodules in the kidneys and lung and associated with pneumonia (without further specifications) was observed once. Paratuberculosis (*Mycobacterium avium* subsp. *paratuberculosis*) was diagnosed once as well, in an young adult male from which only selected organs were submitted (liver, lung, part of large intestines and lymph nodes); necropsy findings were limited to severely enlarged lymph nodes (Figs 4D and 5A) but severe diarrhea was mentioned in the case history. Listeriosis (*Listeria* sp. isolated from abdominal fluid) was documented in one case with aspiration pneumonia and tubulonephrosis; the brain was not investigated. Necrobacillosis (*Fusobacterium necrophorum*, partly in association with pyogenic organisms) characterized by necrosis of the oral mucosa or/and the tongue and /or multiple abscesses in inner organs, especially liver and lung, was observed in five roe deer from all age categories. Enteric clostridial infections (*Clostridium* sp. or *C*. *perfringens*) were diagnosed 11 times, in most cases in association with diarrhea (n = 8). Furthermore, *Clostridium* sp. was found in association with infected wounds (n = 3). Infections with *Escherichia coli* (n = 31) were observed in roe deer that were considered to have died of septicemia or pneumonia.

Additionally, bacterial pneumonias were considered to be a predisposing cause for eight cases of predation by dogs. Finally, a large number of additional bacterial organisms were isolated from roe deer samples (Table 2), however, their potential significance in the disease process was not further mentioned in the report and remains questionable.

**Table 2:** Microorganisms detected by bacteriological and mycological examination in samples from dead free-ranging roe deer examined at the Centre for Fish and Wildlife Health, Switzerland, 1958–2014.

		Associated with main disease	Total numbers detected
**Bacteria**			
Gram (+)			
	*Actinomyces* sp.	2	2
	*Aerococcus viridans*	1	1
	*Bacillus* sp.	1	1
	*Clostridium* sp.	3	3
	*Clostridium perfringens*	11	21
	*Corynebacterium pseudotuberculosis*	1	1
	*Dermatophilus congolensis*	3	3
	*Enterococcus* sp.	0	2
	*Erysipelothrix rhusiopathiae*	2	2
	*Listeria* sp.	1	1
	*Staphylococcus* sp.	17	27
	*Staphylococcus aureus*	6	27
	*Streptococcus* sp.	35	50
	*Streptococcus bovis*	0	1
	*Streptococcus suis*	0	1
	*Trueperella pyogenenes*	47	71
Gram (-)			
	*Actinobacillus* sp.	2	3
	*Aeromonas* sp.	0	4
	*Bordetella* sp.	0	1
	Coliform bacteria	0	11
	*Escherichia coli*	31	95
	*Fusobacterium* sp.	1	1
	*Fusobacterium necrophorum*	6	6
	*Hafnia alvei*	1	1
	*Klebsiella moblis*	1	1
	*Klebsiella pneumoniae*	0	1
	*Manheimia haemolytica*	11	11
	*Manheimia granulomatis*	2	2
	*Manheimia varginea*	1	1
	*Moraxella* sp.	0	5
	*Morganella morganii*	1	1
	*Mycoplasma* sp.	0	1
	*Neisseria* sp.	2	2
	*Pasteurella* sp.	2	12
	*Pasteurella multocida*	14	20
	*Pasteurella septica*	0	2
	*Salmonella enterica sp enterica*	1	1
	*Salmonella* Typhimurium	1	1
	*Yersina pseudotuberculosis*	5	5
Acid-fast			
	*Mycobacterium avium* subsp. *paratuberculosis*^*1*^	1	1
other			
	Mixed bacterial flora	5	41
	Inconclusive or negative	54	361
**Fungi**			
	*Aspergillus* sp.	20	20
	*Aspergillus fumigatus*	3	3
	*Mucor* sp.	4	4
	*Mucor pussilis*	1	1
	Not further specified	8	8

If not otherwise specified diagnosis is based on culture results.

^1^PCR colon feces.

Fungal infections were mostly observed in the lungs (pneumonia) and due to *Aspergillus* sp., or in the head in the form of actinomycosis-like proliferations of soft tissue and bone and due to infections with *Aspergillus* sp. (n = 23) or *Mucor* sp. (not septate fungi, n = 5, 1/5 further characterized as *Mucor pucillis*).

Hypertrophic osteopathy of the metacarpal and metatarsal bones with an exuberant proliferation of cortical bone with coraliform osteophytes, described in the literature as presumably secondary to intrathoracic voluminous neoformations (also referred in the past with more general terms including: hypertrophic osteoarthopathy, digital clubbing, acropathy, or Marie’s disease) [49,50] was observed repeatedly during the study period (n = 14) (Figs 4E and 5E). The associated lung lesions were most often fungal pyogranulomas (n = 7, *Aspergillus* sp.). In fewer cases there were large suppurative collections of bacterial etiology (n = 2, *Streptococcus/Staphylococcus* sp., *Fusobacterium* sp.) or the etiology was unknown (n = 3). In two cases the association with lung lesions was merely suspected because only the legs were sent for examination.

#### Parasitic infections

**Fig 6:**
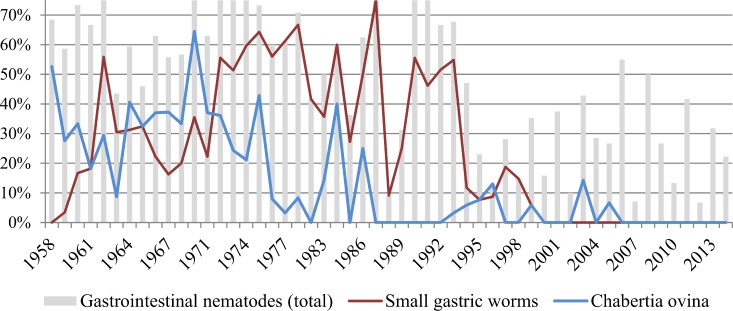
Submitted roe deer with documented gastrointestinal nematodes over time. Percentages correspond to the number of cases with reported gastrointestinal nematodes in relation to the total number of investigated carcasses, independently of the investigation methods (necropsy, washouts, coprology, mucosal scraping). Grey bars: total of gastrointestinal nematodes. Red line: small gastric worms. Blue line: *Chabertia ovina*.

Parasitic infections categorized as cause of death or disease affected mainly the gastrointestinal tract and were associated with local inflammation of the stomach and/or intestinal mucosa and in some cases with diarrhea. In fewer cases the respiratory tract was also affected and the lesional picture consisted of granulomatous pneumonia (Fig 5E). When both organ systems were affected, a diagnosis of multisystemic endoparasitosis was made (Table 1). An overview of the documented parasites is given in Table 3. The reported parasitofauna did apparently not differ between roe deer with and without endoparasitosis as the main diagnosis. There were apparent changes of parasite occurrence (Fig 6) and in the frequency of specific parasitic main diagnoses over time: Small gastric worms and *Chabertia ovina* were documented and interpreted as cause of morbidity or mortality only until 1995, and parasites were mentioned as cause of diarrhea only up to 1998. Nevertheless gastrointestinal and, less commonly, lung endoparasitosis were regularly considered to have predisposed the deer for the development of other diseases, either by weakening the animal or by creating a portal of entry for pathogens.

**Table 3:** Parasites detected either by parasitological investigation or at necropsy in free-ranging dead roe deer submitted to the Centre for Fish and Wildlife Health, Switzerland, 1958–2014.

Affected organs/tissues:		Numbers detected
**Gastrointestinal tract**		
	Gastrointestinal nematodes^1^	658
	*Trichuris* sp.	242
	*Nematodirus* sp.	154
	*Moniezia* sp.	28
	Coccidia^2^	183
**Abdominal cavity**		
	*Cysticercus* sp.	17
	*Setaria* sp.	9
**Liver**		
	*Dicrocoelium* sp.	90
	*Fasciola* sp.	6
**Lung**		
	Small lungworms (Protostrongylidae)^3^	235
	*Dictiocaulus* sp.	160
	Lungworms indet.	131
**Skin**		
	*Trichodectidae*	52
	*Lipoptena cervi*	87
	*Cephenemyia st*.	115
	*Demodex* sp.	1
**Muscle**		
	*Sarcocystis* sp.	87

^1^When specific additional information was provided, this included: gastric worms, small gastric worms; *Trichostrongylidae*, *Hemonchus contortus*, *Trichostrogylus* sp., *Ostertagia* sp.; strongyles, *Chabertia ovina*, *Oesophagostonum* sp.; *Strongyloides* sp. or *Capillaria* sp.

^2^When further information including gender or species was provided, this included: *Eimeria* sp., *Eimeria capreoli*, *Eimeria ponderosa*.

^3^If further information including gender or species was provided, this included: *Capreocaulus capreoli* and *Muellerius* sp.

Infestation with ectoparasites were regularly reported (Table 3) but were considered as the main cause of disease only in isolated cases, including biting lice (*Trichodectidae*.; n = 9) and demodectic mange (*Demodex* sp.; n = 1) associated with alopecia, as well as one case presenting multiples cutaneous and muscular abscesses due to warble flies (*Hypoderma diana*).

Another recurrent parasitic infection was the infestation with botfly larvae (*Cephenemyia stimulator*) in the nasal cavity and the pharynx from April through June, in accordance with the fly biological cycle [48]. It was documented in 140 of the submitted animals and considered mainly to be a secondary finding but in 10 cases the larvae were seen as the main cause of death or disease; presumptive death by asphyxia was specifically mentioned for four of these 10 cases. In six cases botfly larvae were seen as predisposing factor for pneumonia (n = 4) or, as mentioned above, for the development of bacterial meningoencephalitis (n = 1) or actinomycosis-like lesions (n = 2).

Sarcocystosis (*Sarcocystis* sp.) was regularly observed (n = 85) at histological examination of muscle tissues (mainly heart or eye muscle) and classified as a secondary finding. However, in two hunted roe deer from which only a meat sample was submitted for examination, generalized severe sarcocystosis was considered to be the main diagnosis. The first case was submitted because of unusually soft meat consistency and symptoms of gastrointestinal disease in a consumer; no obvious macroscopic changes were noticed at examination in the laboratory. In the second case there were macroscopically visible light tan elliptic foci measuring up to 3mm scattered throughout the musculature.

#### Viral infections

Viral infections were suspected on the basis of pathological changes but confirmed by PCR in one case only. They included three cases of malignant catarrhal fever, twice not confirmed and once conclusively diagnosed thanks to the detection of OHV2 DNA by PCR. In this confirmed case, gross lesions consisted of conjunctival reddening, lung and meninges congestion; histologically a necrotizing vasculitis in the eye and brain were observed (Fig 5B). A lymphosarcoma presumably due to bovine leukemia virus infection was recorded twice within the same year (1994) but not further investigated; diagnosis was based on general lymphadenomegaly with neoplastic proliferation of lymphatic cells. Papillomatosis (roe deer papillomavirus) was suspected once on the basis of the presence of integument lesions consistent with fibropapillomas with amophophilic intranuclear cellular inclusions. In one case with non-suppurative polyoencephalitis, a viral infection was suspected; bornavirus and rabies virus tests were negative and further etiological investigations were not performed.

#### Non-infectious causes

Main diagnoses of noninfectious origin consisted mostly of traumas (61%, n = 371 of 607), including blunt trauma due to traffic accidents (n = 154), predation (n = 142), gunshots (n = 44; including illegal shots) and penetrating wounds compatible with antler-induced injuries (n = 9). Among predation cases, identified or presumptive predators were dogs (n = 76), foxes (n = 25), unspecified Canidae (n = 15), lynx (n = 14), and an unspecified bird of prey (n = 1); in 11 cases the predator could not be identified. Factors considered possible predisposing conditions to predation were documented in 42% of dog, 36% of fox and 21% of lynx prey and consisted either of overall poor general body conditions associated with endoparasitism (n = 16), pneumonia (n = 5) or infected wounds (n = 3) or in noninfectious pathologies including prior trauma (n = 13), acquired or congenital malformations (n = 3) or neoplasms (n = 1). Predation by fox and dogs were present throughout the study period, whereas the first lynx predations were recorded in 1973. Additionally, one animal suspected to have died of a blunt trauma in 2007 was partly consumed by a wolf, as demonstrated by genetic analysis.

Myopathies following captures (n = 11) or chasing by dogs (n = 2) included all known forms of capture myopathy [51–53]: peracute shock syndrome (n = 1), ataxia (n = 7), rupture of gastrognemic muscles with hemoglobinuria (n = 1) and ataxia after renewed stress in capture facilities (n = 1). Histology was performed on nine of these 13 cases. Reported lesions were unevenly colored muscle fibers or non-specified changes described as acute muscle degeneration, and in the case of the peracute shock: severe brain and lung edema together with a severe catarrhal bronchitis and bronchiolitis (Fig 5D) were observed. Additionally, the suspicion of physical exhaustion after being chased by dogs (n = 35) was a diagnosis regularly made until the 1980’s. The diagnosis was based on the observation of a “spotted heart”, congestion and sometimes edema of inner organs (lung, heart, or not specified).

Alimentary disorders (n = 50) were partly attributed to altered digestive physiology (rumen acidosis, bloating) in wintertime and suspected to be the consequence of anthropogenic inappropriate food or feeding procedures (n = 14). Up to 1977 food intoxication (n = 19) was regularly suspected based on various pictures mostly including hyperemic gastrointestinal segments, organ congestion and edema (not further specified). In 1991 the suspicion of 00-colza intoxication was reported three times based on the detection of hemolytic anemia (n = 2) or hemosiderosis in the liver (n = 1) and in 1993 the 00-colza intoxication issue was investigated in the framework of a dissertation [54]. The etiological role of colza could not be elucidated and afterwards this diagnosis did not appear anymore in the dataset although cultivation of colza has virtually doubled during the past 15 years in Switzerland [55].

Neoplasms (n = 32) of different origins were documented throughout the entire study period (see Table 4). Beside the primary tumor, metastases were documented in approximately one third of the cases (n = 12), mainly in lung, liver or spleen. Neoplasms were observed in adult individuals with the exception of three juvenile roe deer with lymphosarcoma (n = 2) (Fig 5C) or undetermined neoplastic lymphadenomegaly (n = 1), and six animals of unknown age. Finally, two females with fibroma and fibrosarcoma, respectively, presented a wigged head.

**Table 4:** Tumors observed in free-ranging dead roe deer submitted to the Centre for Fish and Wildlife Health, Switzerland, 1958–2014.

Primary affected organ	Neoplasms
**Lymph nodes** (n = 11)	Lymphosarcoma (n = 8)
	Histiocytic sarcoma (n = 2)*
	Undetermined
**Head** (n = 10)	Spindle cell sarcoma
	Melanoma*
	Ossifying fibroma
	Fibroma/fibropapiloma
	Osteosarcoma (n = 2)
	Fibrosarcoma
	Round cell tumor (brain)
	Carcinoma of the salivary gland*
	Undetermined
**Ovary** (n = 3)	Carcinoma/teratoma*
	Granulosa cell tumor*
	Undetermined
**Skin of body or limbs** (n = 3)	Fibroma
	Fibrosarcoma*
	Fibropapilloma
**Lung** (n = 3)	Carcinoma (n = 3)
**Liver** (n = 2)	Cholangiocarcinoma
	Hepatocellular carcinoma*
**Kidney** (n = 2)	Renal adenocarcinoma
	Adenoma
**Uterus** (n = 1)	Undetermined

If the number of cases was not specified, the neoplasm was a single occurrence.

*described metastatic spread.

Numerous malformations affecting different organs and systems including teeth, jaw, eyes, bones, claws, esophagus, heart and kidneys were observed (Table 5). Cystic kidneys were regularly reported (n = 26) but, with the exception of two cases, one with additional cysts in the lung and another one with particularly severe kidney lesions, they were considered to be secondary findings.

**Table 5:** Malformations reported in free-ranging dead roe deer submitted to the Centre for Fish and Wildlife Health, Switzerland, 1958–2014.

Affected organ/tissue		Malformation
**Skeletomuscular system**		
	Jaw	Uni- (n = 2) and bilateral (n = 1) brachignatia inferior
		Unilateral upper jaw malocclusion
		Prognathia superior
		Anomaly of the jaw (n = 2)^1^
	Teeth	Oligodontia (only P2 in upper jaw)
		Molar misalignment in upper jaw
	Skull	Right deviation of the nasal bone
		Splintered antlers
	Skeleton	Micromyelia on all four legs (fawn)
		Spinal curvature (foetus)^2^
	Abdominal wall	Large inguinal hernia (Fig 4F)
	Claws	Horn overgrowth on all four legs (n = 2)
**Nervous systems and eyes**		
	Eyes	Microphtalmia unilateral (n = 2)
		Microphtalmia bilateral^3^
**Urogenitaltract**		
	Kidneys	Polycystic kindeys^4^ (n = 26)
		Hypoplasia^1^
	Genital tract	Hermaphrodite
**Gastrointestinal tract**		
	Oesophagus	Diverticulus (n = 2)^5^
**Cardiovascular system**		
	Heart	Ventricle septal defect^1^

If the number of cases was not specified, the malformation was a single occurrence.

^1^Additional finding, not further described.

^2^ Either kyphosis or lordosis.

^3^With endophtalmitis and retinal dysplasia, predisposing to dog predation.

^4^20 females, 5 males and 1 case of unknown sex.

^5^Leading to aspiration pneumonia.

Other recurrent cases throughout the study period included: orphaned fawns dying of inanition, a diagnosis presumed on the basis of missing milk residual and mucosal ulcerations in the abomasum (n = 25); degenerative diseases such as severely worn out teeth in very old animals (n = 22) and winter starvation (n = 18). Furthermore, there were eight does which died of dystocia associated with uterine torsion (n = 4), uterine prolapse (n = 2, one together with a uterine torsion) and three cases with macerated fetuses. Interestingly, all these reproductive disorders were observed only before 1978.

#### Recurrent pathological or clinical pictures

Overall, in our material pneumonia was the most often recorded disease picture (n = 191), followed by diarrhea (n = 98). Diarrhea was more commonly observed before 1977, i.e. during years with higher numbers of submitted cases, and attributed to infectious (n = 53) or presumed noninfectious etiologies. These noninfectious causes included the so-called “spring diarrhea” (n = 14), alimentary intoxication (n = 4), advanced dental abrasion in old animals (n = 2) and unilateral upper jaw misalignment (n = 1). Diarrhea of unclear etiology (n = 24) and the “spring diarrhea” were mentioned only until 1977. The spring diarrhea was more often observed in the spring (March-May) and believed to be caused by a change in diet as described by Bouvier et al. [11].

Alopecia (n = 19) was another disease entity observed throughout the study period. Beside the above-mentioned ectoparasitic etiology that was identified in about half of the cases (n = 9), one was attributed to Addison disease caused by a bacterial infection of the pituitary gland and in nine other cases the etiology remained undetermined. Interestingly, all animals with alopecia of undetermined origin were adult females, except for one fawn (female as well). From 1992 to 1998, alopecia was attributed to cadmium intoxication and/or zinc deficiency whereas ectoparasites (biting lice; *Trichodectidae*) were not considered as an etiological cause, even if they were present. Additionally, alopecia was described eight times as an additional finding without further indication of the etiology.

Eye lesions were also recurrently observed (n = 66), in the form of keratitis or keratoconjunctivitis. They were related to various etiologies, mostly of infectious origin (n = 43), with a majority of bacterial infections (n = 31). In 11/31 cases, including six fawns, the eye lesions were attributed to a primary trauma, including presumptive fox bites. Other eye disorders of infectious origin comprised mycotic (n = 5), viral (n = 2, MCF infection and a previously undescribed herpesvirus) or unknown etiology (n = 1). Noninfectious eye diseases included trauma (n = 5) unilateral microphtalmia (n = 2) and retrobulbal lymphosarcoma (n = 1). Origin of eye disease remained undetermined in 13 cases. In about a third of the cases (n = 18) other head structures were also involved in the pathological process.

**Fig 7:**
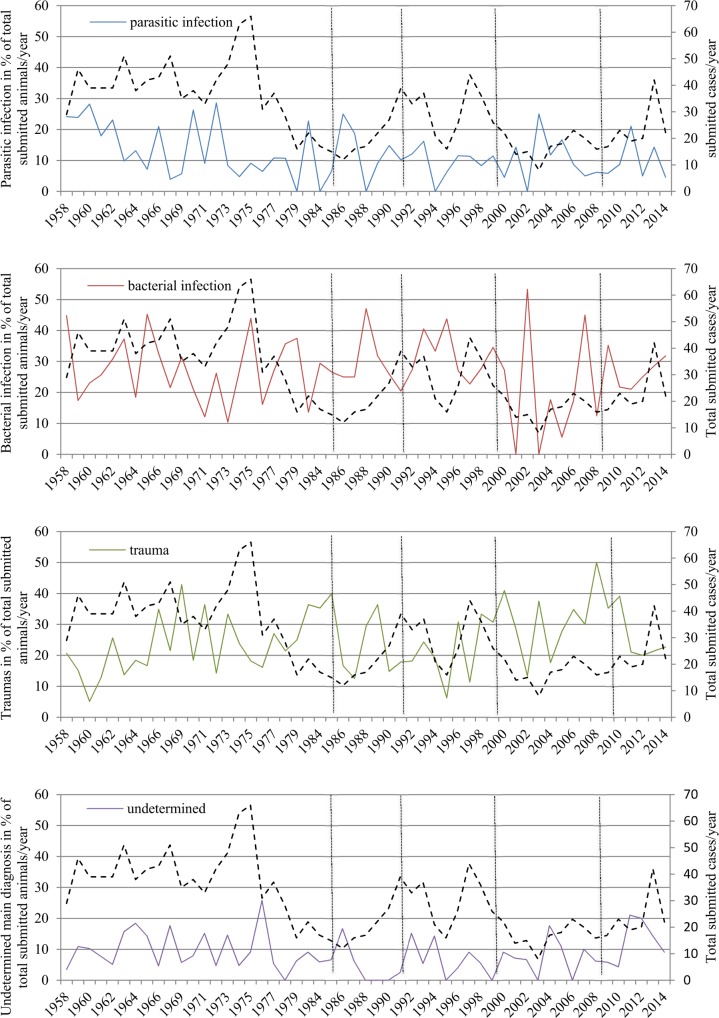
Selected categories of main diagnoses in percentage of total submitted cases per year. (A) Parasitic. (B) Bacterial. (C) Traumatic. (D) Undetermined. Dashed horizontal black lines indicate total number of submitted cases. Vertical dotted lines separate time periods with different main pathologists.

The number and percentage of the observed bacterial and parasitic infections, traumas and of unclear cases strongly fluctuated over the study period but remained roughly within the same range for all time periods, i.e., independently of personnel and methodical changes (Fig 7).

## Discussion

### Causes of death and disease pattern in roe deer

An important goal of this study was to provide an overview of the causes of mortality and morbidity of roe deer observed in the framework of general health surveillance activities over half a century in Switzerland and the Principality of Liechtenstein. We also wanted to assess potential changes in the observed disease pattern that may reflect or be explained by environmental alterations which occurred during that time period. The observed diseases were in accordance with earlier reports from Switzerland and other European countries. Non-infectious causes included traumas of anthropogenic origin (such as traffic accidents and poaching) and of natural origin (including predation and antler punctuation). Cases of predation by dogs and foxes were found over the entire study period, while predation by lynx and detection of wolf DNA on a roe deer carcass appeared for the first time in 1973 and 2007, respectively, which corresponds to the return of these large carnivores to the Swiss regions concerned.

Neoplasms in various organs have been repeatedly described in roe deer (e.g. [56–65]), suggesting that this species is particularly prone to developing neoplastic diseases. However, the most frequently diagnosed tumor in our study was lymphosarcoma, while reports from other countries mention that liver neoplasms is the most frequent form in roe deer [60,66]. Liver tumors in roe deer are characterized by geographical clustering [66] and it has been hypothesized that their etiology is related to the high amount of spruce in the animal diet [67]. This was not supported by our data, as spruce (*Picea abies*) is the predominant tree species in the Swiss Prealps, Alps and Jura [68].

Like for neoplasms, we report anomalies, reproductive disorders and alimentary disorders which have been previously described [9,13,69–73]. Alimentary disorders such as rumen acidosis and bloating are likely only relevant where wild ungulates are actively fed by humans, a practice that has been strongly reduced in Switzerland in the past decades and has even been forbidden in some cantons [74].

Infectious diseases outweighed the non-infectious causes in our dataset but there was no indication of the occurrence of any outbreak of contagious disease in the roe deer population and none of the identified diseases can be considered as a potential threat to the health of the roe deer, domestic animal or other wildlife populations. Observed infectious diseases listed by the OIE or classified by the OIE Wildlife Working Group as additional important diseases of wildlife included paratuberculosis, listeriosis, salmonellosis, caseous lymphadenitis (pseudotuberculosis) and malignant catarrhal fever (all previously reported in roe deer [9,10,12,75–77]). Another listed disease mentioned in the dataset was enzootic bovine leucosis but, as mentioned above, the infectious origin of the lymphosarcoma was merely presumed. Infection with the enzootic bovine leukemia virus seems unlikely as several studies in France and Germany aiming at assessing the role of wild cervids in the epidemiology of enzootic bovine leucosis did not detect antibodies to this virus in roe deer [15,17,78–80]. Besides the zoonoses of the OIE lists mentioned above, *Sarcocystis* sp., *Erysipelotrix rhusiopathiae*, *Corynebacterium pseudotuberculosis* and *Staphylococcus aureus* were four pathogens with zoonotic potential which were also documented in our material, as well as in previous reports [10,11,81,82]. It is noteworthy that all these infections have been observed not only in roe deer but also in other wild species in Switzerland ([43], pers. com. M.-P. Ryser-Degiorgis). Although their sporadic occurrence suggests that the risk of pathogen transmission to other animals and humans is relatively low, it underlines the importance of educating hunters regarding wildlife disease and meat hygiene.

Infestations with gastrointestinal and lung parasites were common and besides being considered a cause of death or disease, they were also often recorded as a secondary finding. The identified parasites were in accordance with data collected by other authors from Switzerland and other countries [11,83–86]. This indicates that endoparasitosis is common in roe deer and not necessarily clinically significant, but it may contribute to the disease process when challenging environmental conditions or other weakening causes occur. Therefore it is questionable as to whether parasitic infections may be considered to be the sole predisposing factor for the main diagnosis, as repeatedly proposed in the necropsy reports considered for this study. We noticed major differences in the detection of gastrointestinal stongylids over time, with most of them being identified only in the early years of the study period. In particular, *Chabertia ovina* and small gastric worms, both documented to be relevant causes of clinical disease in roe deer [87,88] dramatically decreased in the early 1990ies. This lack of detection is likely due to changes in laboratory methods and personnel (see below) rather than to a change in the helminthofauna of roe deer.

Pneumonia, diarrhea and eye disorders of various etiologies, purulent meningoencephalitis (often associated with skull wounds suggestive of territorial fights between males, similarly to cranial abscess disease in white-tailed deer, *Odocoileus virginianus*[89]) and actinomycosis/actinomycosis-like lesions have also been reported in roe deer by other authors [9–11,13] and obviously belong to the typical disease spectrum of this species. By contrast, other authors have apparently not observed alopecia to the same extent as we have, although demodectic mange has been previously reported [90,91]. In our study, alopecia was often associated either with biting lice infestation or else the etiology was unclear. Among these unclear cases, the predominance of old females was striking and warrants closer attention in the future. The diagnosis of Addison’s Disease in one female with this clinical picture suggests that hormonal disorders need to be considered. Finally, the frequency of the diagnosis of winter starvation was much lower in our material than in former observations from Sweden [9,12], a difference that may be explained by the much harsher climate in northern than in central Europe.

### Material quality

This retrospective study also aimed at exploring the value and limitations of the collected material. General surveillance datasets typically give a valuable insight into the health status of a population but do not provide representative data in terms of frequency of occurrence of the diagnosed diseases because they are influenced by a number of field and laboratory factors [92]. In our dataset, which extends over half a century in a country structured into numerous cantons with different laws, languages and traditions, wildlife carcass submissions have strongly varied over time and space and data comparison between geographical regions and time periods is very limited. Nevertheless, the fact that roe deer originated from 23 out of 26 Swiss cantons as well as from the Principality of Liechtenstein shows that the awareness of the possibility to send animals for investigation is present in almost all of the country, which is an essential factor for the early detection of emerging diseases. Furthermore, the lack of animals from the western part of the country (mainly the cantons of Vaud and Geneva) in our study should not be interpreted as a gap in surveillance, as animals from this region used to be systematically sent to a local laboratory (Institute Galli-Valerio, Lausanne; [14,34]). Similarly, the largest part of the carcasses sent to the FIWI originated from the canton of Bern, where the FIWI is located, suggesting that the proximity of the laboratory strongly influences the motivation to submit material for analysis. Furthermore, cantons with professional game wardens sent more animals for investigation than others, showing that educated field partners fully dedicated to wildlife management play a crucial role in wildlife health surveillance. Finally, the drop of submissions in the canton of Bern in the 1980ies indicates a change of intensity or strategies of wildlife health surveillance by field partners during that time period. Similarly, the number of roe deer examined at the Institute Galli-Valerio has dropped after 1996 due to a budget restriction requiring a stronger case selection [14].

In contrast to our dataset, the official Swiss hunting statistics (which has been conducted since 1968 and includes not only hunting bags but also animals found dead or culled due to disease signs) [38] shows that the largest part of reported deaths in roe deer is due to non-infectious causes of anthropogenic origin even when hunting is excluded. Similarly, in the hunting statistics predation by dogs accounts for as many death as the disease category, and predation by large wild carnivores has increased over time, which is in accordance with the increasing population of Eurasian lynx and wolves but strongly differs from the pattern of causes of mortality in our dataset. Furthermore, fox predation was poorly represented both in our material and in the hunting statistics, although it is one of the major causes of mortality in roe deer fawns [93]. These contrasting figures may be explained partly by the chance to detect a carcass (underestimation in both datasets) and partly by the motivation of the finder to submit it for further investigations (underestimation in our dataset only). A study on radio-collared lynx has shown that animals killed in relation to legal human activities are more likely to be found than sporadic cases of infectious disease [94], an observation that is expected to be valid for other species. By contrast, cases of predation are usually not found when prey size is small and/or complete carcass consumption rapidly achieved [95]. Furthermore if a carcass is detected, field partners’ motivation to submit it is higher in case of unusual or unclear mortality than in the presence of known entities such as traffic kills or diseases characterized by typical macroscopic lesions. In line with this, general health surveillance in hunted animal species in Switzerland aims at elucidating unclear cases of death and at detecting emerging causes of mortality and does not include the submission of the totality of the carcasses found in the field [34].

Laboratory methods and personnel have changed repeatedly over the past decades while standards for diagnostic quality have gradually increased (e.g. introduction of accreditation standards). Interestingly, our dataset illustrates that these changes have not only resulted in improved diagnostic performance: While the use of new tools such as PCR have permitted to confirm the involvement of specific pathogens such as ovine herpes virus or *Mycobacterium avium* subsp. *paratuberculosis*, the replacement of traditional parasitology methods by new standards has caused a loss of information. In contrast to the parasitological necropsies, washouts and mucosal scrapings performed in the past, the coprology method allows only egg detection and identification, which is not sufficient to distinguish between different species of gastro-intestinal strongylids. This loss of accuracy in parasite identification did not cause a decrease of the total diagnosed parasitic infections among the main diagnoses but small gastric worms and the associated “epidemic stomach disease” disappeared from the dataset. Furthermore, the observed shift from bacteriology and parasitology (both decreasing) to histology (increasing; Fig 3) did not affect the total amount of diagnosed parasitological or bacteriological etiologies (Fig 7), indicating that histology may at least partly compensate the other methods. Another advantage of histology is that it can assess associations between pathogen identification and the observed tissue lesions.

Additional factors which have likely influenced the disease pattern over time are the experience and skills of the pathologists in charge. With each personnel change there has been a potential loss of knowledge which cannot be evaluated based on the existing written records. For example, it is unclear as to whether the diagnoses of food intoxication and stress syndromes related to pursuits by dogs were well founded (history, previous experiences) or merely conjectured. Furthermore, the fact that *Chabertia ovina* and small gastric worms have disappeared from the dataset are likely not only due to changes in the laboratory methods but also to a loss of knowledge over time. This hypothesis is supported by recent studies which have documented these parasites in roe deer from other countries [83,84,86]. Thus, targeted investigations are required to assess the current occurrence of gastric helminths and their importance in pathological processes in roe deer in Switzerland. The reasons for the disappearance of diarrhea of unknown etiology and of the “spring diarrhea” are unclear as well. It may be related to an attribution of these diarrheas to infectious etiologies in recent years (parasites, clostridia); which of these interpretations is correct warrants further investigations. Another possibility is that the “spring diarrhea” is well-known by field partners, who do not send such cases in anymore. The drop of cases with this diagnosis corresponds indeed to the drop of total submitted cases in the late 1970’s, which most likely corresponded to a change in the surveillance strategy (canton of Bern). Changing disease awareness of field partners and lab workers can seriously influence case submission and the achieved diagnoses. Among others, the disease pattern in the dataset may be strongly influenced by the fact that specific diseases come into or disappear from the focus of the preoccupations of the health authorities and/or the media [92]. In accordance with this, the two suspected cases of enzootic bovine leucosis were mentioned during the first year of the national eradication program for this disease in Switzerland [96].

Virological investigations were rarely performed over the entire study period and viral diseases were possibly underdiagnosed. However pathological findings were rarely suggestive of a viral infection. Rabies was not represented in our dataset although cases have been diagnosed in roe deer in Switzerland up to the 1990’s [97]. For safety reasons, rabies diagnostic is performed before necropsy by the Swiss Rabies Center and, in contrast to animals which test negative, those which test positive are not forwarded for complete necropsy. Consequently our material did not reflect the disappearance of rabies in roe deer following eradication [98].

Similarly, a toxicological investigation was performed only once, and toxic etiologies, whether as a main diagnosis or as a predisposing factor (e.g. pollutants) [99], may well have been missed. A study performed in Switzerland in the 1980’s—1990’s on the chronic exposure of roe deer to poisonous heavy metals, chlorinated hydrocarbons and polychlorinated biphenyls revealed a massive drop of the lead content in roe deer tissues after the implementation of lead-free fuel in the mid 1980’s [pers. com. M.-P. Ryser-Degiorgis]. This improvement does not seem to have significantly influenced the disease pattern in roe deer but the data illustrate that the investigation of chronic subclinical intoxications are of particular interest when assessing the potential effect of environmental alterations.

A notable difficulty encountered in this study was that the old reports were partly incomplete, in a state of poor conservation (vanishing ink and/or missing documents), hand-written and difficult to read, or insufficiently detailed as regards the description of post-mortem observations or the justification of the diagnosis. Additionally, the archive of paraffin blocks and histological slides was incomplete, making a retrospective evaluation of the accuracy of doubtful diagnoses impossible.

Finally, comparisons of our data with those obtained in other countries were hampered not only by the lack of representativeness of the investigation material, but also because data have been classified in different ways. Difficulties are encountered particularly when authors mix morphological diagnoses and etiologies when calculating percentages and do not clearly define the categories and criteria they use for classification.

## Conclusion

Despite the many biases in the available material, this long term evaluation provided valuable information about the occurrence of diseases and the associated pathological findings in free-ranging roe deer. Comparison with old Swiss studies and reports from other countries suggest a globally uniform disease pattern. The analysis and publication of such baseline data is essential for the local and global understanding of free-living animal populations [100]. Importantly, it can serve for the education of people involved in the surveillance of wildlife diseases, leading to a better disease awareness and human health prevention. Furthermore, our study indicates that professional game wardens are an added value to wildlife health surveillance programs and shows that not only changes in methodology but also changes in personnel may result in a loss of knowledge. To facilitate comparisons in space and time we recommend the systematic collection of metadata for all submitted cases, keeping record of the cases’ history and all the details of the investigations (including methods and results) as well as the histological slides or paraffin-embedded tissues. Archiving of frozen tissue specimen should also be considered whenever the required storage space and manpower is available. Furthermore, we encourage performing such data analyses on a more regular basis, as changes in methods and personnel are inherent to long time periods. Importantly, records and classification of diseases and causes of death need to be performed in a harmonized way.
